# Disrupted functional connectome in a rodent model of autism during social isolation

**DOI:** 10.3389/fncir.2025.1525130

**Published:** 2025-05-14

**Authors:** Robert Gergely Kemecsei, Szizel Dániel-Papp, David Barnabas Balazs, Estifanos Ghebrihiwet Tewelde, Andras Csillag, Gergely Zachar

**Affiliations:** ^1^Department of Anatomy, Histology, and Embryology, Faculty of Medicine, Semmelweis University, Budapest, Hungary; ^2^HUN-REN Institute of Experimental Medicine, Budapest, Hungary; ^3^Department of Ethology, Eötvös Loránd University of Sciences, Budapest, Hungary

**Keywords:** autism spectrum disorder, valproic acid, VPA, social isolation, social decision making network, stress regulating network, functional connectivity

## Abstract

Autism spectrum disorder (ASD) is associated with disruptions in social behavior and the neural circuitry behind it. Very little data is available on the mechanisms that are responsible for the lack of motivation to reunite with conspecifics during isolation. It is as important to investigate the neural changes that reduce motivation to end social isolation, as those underlying the reactions to social stimuli. Using a rodent model of prenatal valproic acid (VPA) exposure, we investigated how social isolation affects the neural activation of key brain nuclei involved in social processing and stress regulation. Juvenile male C57BL/6 mice were treated prenatally with VPA or saline (CTR) and subjected to 24 h of social isolation from their cage mates, with neural activity assessed via c-Fos immunohistochemistry. Based on correlational activations we reconstructed and analyzed the functional connectome of the observed brain regions. Control animals exhibited elevated c-Fos expression in the regions central to the mesolimbic reward system (MRS), social brain network (SBN), and stress-related networks, with the interpeduncular nucleus (IPN) at the core, compared to VPA-treated animals. Functional network analysis revealed a more widespread but less specific pattern of connectivity in VPA-treated animals. These findings suggest that prenatal VPA exposure disrupts certain neural circuits related to social behavior and stress regulation, offering an insight into the altered perception of social isolation in ASD models, and highlighting potential therapeutic targets.

## 1 Introduction

Autism spectrum disorder (ASD) is a neurodevelopmental condition characterized by deficits in social communication, restricted interests, and repetitive behaviors (Hodges et al., 2020). Its etiology involves a complex interplay between genetic and environmental factors, with gestational exposure to valproic acid (VPA) being particularly implicated (Deth et al., 2008; Karimi et al., 2017). Rodent models of VPA exposure replicate certain aspects of ASD, notably diminished social preference, which is evident in sociability tests such as the three-chamber test (Lord et al., 2000; Chaliha et al., 2020). On the other hand, autistic patients are affected differently not just by social stimuli (Schreibman and Lovaas, 1973; Buitelaar, 1995; Dichter and Belger, 2007) but also by the lack of these, namely social isolation (Lloyd-Fox et al., 2013; Smith and Sharp, 2013). Reports from parents of autistic children during the Covid-19 lockdown suggest that the socially less challenging environment often made their child “calmer” and “happier” (Mumbardó-Adam et al., 2021), however, as most of the studies reported it, the long term social isolation (from one and half up to 6 months) resulted in a worsening of the symptoms of autistic children (increased irritability, anxiety, and emotional dysregulation). This was probably due to the lack of the improving effects of social interactions (Lloyd-Fox et al., 2013; Smith and Sharp, 2013; Ahmed et al., 2024).

Mice, like humans, are social creatures that typically live in groups, and social isolation is known to induce stress (Gutman and Nemeroff, 2003; Lukkes et al., 2009a, 2009b; Brandt et al., 2022). However, most studies on VPA-treated animals have focused on its effects on social interactions, preferences, and memory (Di et al., 2022; Zheng et al., 2023; Ukezono et al., 2024), while little is known about how these animals respond to social isolation. Since autism and its animal models are defined by diminished sociability (Crawley, 2007; Kim et al., 2011; Di et al., 2022), it is likely that autistic mice perceive and react to isolation differently than control mice, similarly to humans.

We hypothesized that differences in social preference and responses to isolation likely reflect the impact of VPA on the development of several brain regions that shape social behavior. One of such networks is the “social behavior network” (SBN), a neural circuit responsible for integrating social functions such as reproduction, aggression and affiliative behaviors in mammals (Newman, 1999; O’Connell and Hofmann, 2011; Sato et al., 2023). Beyond reproduction-related behaviors, animals evaluate environmental (including social) stimuli to generate appropriate responses. The nodes that make up the SBN are the preoptic area (POA), ventromedial hypothalamus (VMH), anterior hypothalamus (AH), periaqueductal gray/central gray (PAG), medial amygdala (meAMY), lateral septum (LS), and the bed nucleus of the stria terminalis (BNST). Each of these brain regions has proved to be important in regulating both reproductive and aggressive behaviors in mammals (O’Connell and Hofmann, 2011; Falkner and Lin, 2014; Tovote et al., 2015; Hashikawa et al., 2016; Fischer and O’Connell, 2017; DeAngelis and Hofmann, 2020). The POA and PAG, while commonly analyzed as unified regions, encompass distinct subregions with divergent functional roles. The POA consists of lateral (lPOA) and medial (mPOA) divisions that contribute differently to social behavior: the lPOA is associated with adaptive, goal-directed responses via modulation of dopaminergic circuits (Gordon-Fennell et al., 2020), whereas the mPOA is implicated in parental care and aggression-related activity patterns (Zhong et al., 2014; Sato et al., 2020). Similarly, the PAG is composed of functionally distinct columns, with the dorsomedial and dorsolateral subdivisions (dmPAG, dlPAG) primarily engaged in social behaviors, such as intermale aggression and social fear regulation. These regions receive monosynaptic inputs from the VMH and process socially relevant threat cues (Kincheski et al., 2012; Li et al., 2025). In contrast, the lateral and ventrolateral PAG (lPAG, vPAG) mediate non-social defensive responses, including immobility and autonomic inhibition, typically activated by nonsocial threats (Fanselow, 1994; Brandão and Lovick, 2019). This anatomical and functional partitioning underscores the importance of subregional analysis in delineating social versus generalized stress-related circuits. LS and BNST are also parts of the “mesolimbic reward system” (MRS), a key neural network for assessing the salience of various stimuli (Deco and Rolls, 2005; Wickens et al., 2007; Duque-Wilckens et al., 2020; Kelly, 2022). They act as critical junctions between the SBN and MRS, mediating the relevance of social stimuli and generating adaptive behaviors such as aggression or affiliative responses (O’Connell and Hofmann, 2011; Duque-Wilckens et al., 2020; Kelly, 2022). MRS is a key neural network, where the salience of stimuli is evaluated (Deco and Rolls, 2005; Wickens et al., 2007). The dopaminergic pathway formed by projections of midbrain dopamine neurons from the ventral tegmental area (VTA) to the nucleus accumbens (NAcc) is a key element of the mesolimbic system, responsible for reward evaluation. The conventional reward system also includes the basolateral amygdala (blAMY), LS, ventral pallidum (VP), caudate-putamen (CPU), hippocampus (HIP), and BNST (Cardinal et al., 2002; Kami et al., 2018; Takahashi et al., 2019; Wulff et al., 2019; Morel et al., 2022; Wei et al., 2025). Given the phylogenetically ancient nature of the functional contexts in which animals behave (i.e., mate choice, male–male aggression, foraging, etc.), it is reasonable to hypothesize that the mesolimbic dopamine system plays a conserved role in reinforcing these behaviors in vertebrates (O’Connell and Hofmann, 2011). The anterior cingulate cortex (ACC) plays a crucial role in social decision-making, attentional control, and emotional regulation, all of which are essential for adaptive social behavior (Schneider et al., 2020). It is functionally connected to the mesolimbic reward system (MRS) and modulates dopamine-dependent reinforcement learning, particularly in response to social stimuli (Schneider et al., 2020). Given its role in dopaminergic regulation and its integration of social and affective information, the ACC was included as a key node within the MRS to better understand its contribution to social behavior modulation. The overlapping SBN and MRS consists of the so-called social decision-making network (SDMN), which appears to be an evolutionary conservative neural substrate that regulates all types of social behaviors (O’Connell and Hofmann, 2011; Wei et al., 2025).

Besides SDMN, the habenulo-interpeduncular (Hb-IPN) axis is also involved in mood-associated conditions, serving as a pathway for signaling stress evoked by social and other conditions (Mirrione et al., 2014; McLaughlin et al., 2017; Ables et al., 2023). This pathway, along with the lateral habenula (lHb), integrates signals from SDMN, playing a crucial role in modulating reward-related behaviors (Hikosaka et al., 2008; Boulos et al., 2017). The medial habenula (mHb) receives projections from several nodes of the SDMN, including POA, LS, and NAcc. Similarly, lHb also receives inputs from the SDMN, including meAMY, VP, and BNST (Boulos et al., 2017). On the contrary, lHb also acts on other SDMN nodes, including VTA and PAG, via the rostromedial tegmental nucleus (Hikosaka et al., 2008; Graziane et al., 2018). The Hb sends its densest efferent projections to the mesencephalic IPN through the core of the fasciculus retroflexus (Herkenham and Nauta, 1979; Okamoto et al., 2021), which, in turn, sends efferents to a variety of midbrain and hindbrain structures implicated in regulating affective states. Such structures are the hippocampus, lateral hypothalamus, VTA, septum, and POA (Hayakawa et al., 1981; Shibata and Suzuki, 1984; Morley, 1986; McLaughlin et al., 2017). Beyond the Hb-IPN axis, additional structures modulate social stress responses. The zona incerta (ZI) integrates sensory and limbic information to regulate defensive arousal and has been shown to modulate different components of anxiety (Li et al., 2021). The lateral hypothalamic area (LHA) contributes to stress-related behaviors, including active fear responses such as jumping and escape (Li et al., 2018). Within the amygdala, subregions such as the central nucleus (CeAMY) and basomedial nucleus (bmAMY) also modulate social stress reactivity and sensitivity to social context (Ventura-Silva et al., 2013; Ritger et al., 2023). Finally, the interfascicular nucleus (IF), located in the medial aspect of the VTA, has been identified as a stress-sensitive node that is highly responsive to social challenges and contributes to stress-induced neuroplastic adaptations (Tang et al., 2014).

### 1.1 Aim of study

In the present study, we systematically mapped brain region activation following 24 h social isolation by labeling c-Fos protein in the SDMN as well as in other regions involved in social stress processing, such as Hb-IPN axis (Sugama et al., 2002; Benekareddy et al., 2018; Xu et al., 2018; Ogawa and Parhar, 2022). Previous studies have shown that even a single day of social isolation significantly elevates c-Fos expression in the Hb system of rodents (van Kerkhof et al., 2013; Stanisavljević et al., 2020). This increased activity is likely accompanied by distinct patterns of brain activation in several regions of the SDMN, which may serve as a basis for differentiating VPA-treated mice from control mice. Beyond its role in acute social stress processing, growing evidence links the habenula to the pathophysiology of autism spectrum disorder. In ASD models, altered habenular excitability and disrupted glutamatergic signaling within the lateral habenula have been shown to contribute to social deficits and motivational impairments (Murru et al., 2021; Xu et al., 2023). Moreover, structural alterations of the habenula have been reported in individuals with ASD, further underscoring its relevance in social behavior regulation and autism-related neurocircuitry (Germann et al., 2021). Since idiopathic autism and embryonic VPA exposure affect multiple neural pathways rather than an isolated brain region, we applied a network-based approach to examine the broader functional organization of affected circuits (van Kerkhof et al., 2013; Nagode et al., 2017; Ádám et al., 2020; Dorsey et al., 2024). To achieve this, we constructed a functional connectome based on correlational activation patterns across brain regions (Wheeler et al., 2013; Stefaniuk et al., 2023; Kotlarz et al., 2024). This method enabled us to explore alterations in the broader neural network structure, rather than focusing solely on localized changes.

To support region-specific interpretation of neuronal activation, four functionally distinct brain regions were included as controls (with presumably no direct influence on social behavior): the dorsomedial hypothalamus (DMH), the arcuate nucleus (ARC), the Edinger–Westphal nucleus (EW), and the dentate gyrus (GD). The DMH was selected based on its broad integrative role in autonomic and behavioral regulation within the hypothalamus (DiMicco et al., 2002; Kataoka et al., 2013). The ARC serves as a key neuroendocrine and metabolic center, providing a reference for activation patterns in nuclei involved in homeostatic regulation (Song and Choi, 2023). The EW, particularly its centrally projecting subpopulation, represents a functionally divergent midbrain structure, allowing comparison to regions with different neurochemical and anatomical profiles (Kozicz et al., 2011). Finally, GD, the primary gateway of cortical input to the hippocampus, was included to represent a general input structure with a well-defined role in hippocampal information processing (Hainmueller and Bartos, 2020).

We hypothesize that VPA-treated mice, modeling the autistic phenotype, exhibit diminished stress-related neural activation and altered SDMN network-wide connectivity in response to social separation compared to typically developing mice. By analyzing these circuits, we aim to deepen our understanding of the mechanisms underlying ASD and identify potential targets for therapeutic intervention.

## 2 Methods

### 2.1 Animals and housing conditions

C57BL/6 mice were maintained on a 12:12 light–dark cycle, with the dark phase beginning at 8:00 AM. Food and water were provided ad libitum. All experiments were conducted at least 2 h after lights-off to ensure alignment with the animals’ active phase. Female mice were housed in pairs, with timed mating achieved by introducing a male for 24 h. Pregnancy was confirmed by monitoring weight gain on day 13.5 of gestation (E13.5) when the VPA and control treatment took place (Kim et al., 2011; Mehta et al., 2011; Kang and Kim, 2015; Zarate-Lopez et al., 2024). Pregnant mice were injected subcutaneously with either Na-VPA (Depakine® 400 mg/4 mL 500 mg/bwkg) (VPA-treated) or volume-matched saline for control groups (CTR). Male pups from 14 different litters were weaned on postnatal day 24 and used for the experiment on postnatal day 31 (n_CTR_ = 10, n_VPA_ = 11). The animal study was approved by the Food Chain Safety and Animal Health Directorate of the Government Office for Pest County, Hungary (PE/EA/926–7/2020). The study was conducted in accordance with the local legislation and institutional requirements.

### 2.2 Three-chamber sociability test

Group level behavioral differences caused by VPA treatment were validated using the three-chamber sociability test on the day of weaning (day 24). The apparatus consisted of three connected transparent Plexiglass boxes (19 × 45 cm) (Kaidanovich-Beilin et al., 2011). In the initial session, mice were habituated to the environment for 5 min. During the social preference session, an unfamiliar control mouse was placed in one terminal chamber, while the other remained empty. Each stimulus mice were used in two tests (one for a VPA and one for a control animal) in randomized order. Such stimulus mice were later incorporated into the social housing groups as companion animals, however, they were never used as experimental individuals. The test lasted 10 min, with data from the first 5 min analyzed, as exploratory behavior and social interactions primarily occurred during this initial period. After this time, animals generally became less active, and their chamber exploration plateaued. The entire session was video recorded and analyzed using Solomon Coder (2024) software. Sociability was evaluated by measuring both the total time spent in each chamber and the average visit duration. The average visit duration was defined as the mean duration of individual visits to a chamber, calculated as the time elapsed between an entry and an exit. The total time spent in a chamber represented the cumulative duration of all visits within the session. The data were statistically compared using the Wilcoxon signed-rank test.

### 2.3 Post-test housing and separation protocol

Following the three-chamber test, animals were cross-fostered into mixed groups of five, including both VPA-treated and control (CTR) mice. The animals in each group were from different litters, and each group consisted of a mix of VPA-treated (2 or 3) and CTR individuals. On postnatal day 31, one mouse from each group was separated for 24 h. While 24 h of social deprivation might appear too long to induce specific c-Fos activation, van Kerkhof et al. (2013) showed that 24 h of isolation caused a higher increase in c-Fos expression in some brain areas than 3.5 h of isolation. The separated mice were placed in a new home cage with fresh bedding, supplemented with a small portion of bedding from their previous cage. The cages of separated mice were kept in the same room but positioned far from their previous cage mates. On postnatal day 32, after 24 h of isolation and at least 2 h into the dark phase, the animals’ active period, mice were anesthetized using isoflurane (Forane®) and transcardially perfused with physiological saline, followed by 4% paraformaldehyde (PFA).

### 2.4 Sample preparation and immunohistochemistry

Brains were stored in 4% PFA and then transferred to 25% sucrose in PBS 1 day before sectioning. After sucrose infiltration, brains were frozen and sectioned at 50 μm using a freezing microtome (Leica SM 2000R). For c-Fos immunohistochemistry. Free-floating sections were rinsed in PBS, and endogenous peroxidase activity was blocked for 20 min at room temperature (RT) using PBS containing 2% H₂O₂. Blocking was performed for 1 h at RT in PBS containing 1% normal goat serum and 0.2% Tween-20, followed by incubation with a polyclonal rabbit anti-c-Fos antibody (1:5000, Abcam, ab190289) in PBS with 1% normal goat serum and 0.02% Tween-20 for 1 h at RT and then overnight at 4°C. On the following day, the slices were incubated for 2 h at RT in a biotinylated anti-rabbit IgG secondary antibody (1:200, Biotinylated IgG, Vector Laboratories, BA-1000), followed by overnight incubation at 4°C in avidin-biotin solution (1:500, ELITE ABC kit, Vector Laboratories) containing 0.01% Tween-20. After rinsing in PBS and Tris buffer (pH 8.0), sections were incubated in NiDAB solution (0.15 mg/mL DAB + 4.25 mg/mL Ammonium nickel sulfate Hexahydrate) for 10 min at 4°C, then developed for 5 min at RT with the addition of 1 μL dense H₂O₂. Following the staining, the sections were mounted onto chrome-gelatin-coated slides, dehydrated through an ascending ethanol series [50, 70, 90, 96, 100% (v/v)], cleared in xylene, and coverslipped using DePeX mounting medium.

### 2.5 Image acquisition and region of interest (ROI) analysis

Images were captured digitally using a Nikon Eclipse E800 microscope equipped with an Olympus DP74 camera. Brain regions were identified based on the SDMN framework (O’Connell and Hofmann, 2011), with the following modifications: The Decision-Making Network was expanded to include ACC, and some of the SDMN regions were further subdivided according to functional differences. Regions associated with social stress and autism were also analyzed. Regions were imaged at the same rostrocaudal coordinates, determined by anatomical landmarks, using 4 × magnification to ensure that the entire region of interest (ROI) was fully visible within a single image. Each ROI was imaged bilaterally, with at least one image taken from each hemisphere per subject, allowing for the analysis of laterality effects. For larger nuclei spanning multiple sections, such as the BNST, multiple images were taken from different slides within the same rostrocaudal coordinate range, ensuring comprehensive anatomical representation (Atlas Thumbnails, 2024). For cell quantification, the entire ROI was outlined based on anatomical references from the mouse brain atlas, and c-Fos + cells were counted within the fully delineated area to maintain consistency across subjects.

### 2.6 Cell counting and statistical analysis

c-Fos positive cells were quantified using the particle analysis function of ImageJ (2024), following background subtraction and Gaussian blur to enhance detection accuracy. A size filter (70–200 pixels) and a circularity threshold (0.85–1.00) were applied, and signals meeting these criteria were considered c-Fos-positive cell nuclei. These validated counts were then used for subsequent statistical analyses. Data were analyzed using the statistical software R. For each brain region, c-Fos-positive cell counts were compared between the experimental groups using a negative binomial mixed-effects model (model parameters: response variable: c-Fos positive cell number; main effect: Embryonic treatment + nested random effects litter, individual) + offset [log(Area of ROI)] (Yu et al., 2022). False discovery rate (FDR) correction was applied to *p*-values using the Holm-Bonferroni method, setting the significance threshold at 0.05. Within subject pairwise comparisons failed to detect any significant inter-hemispheric differences, therefore laterality as a factor was not included in the final model. In cases where the p-value ranged between 0.05 and 0.1, Cohen’s D values were calculated using the “effsize” R-library (Torchiano, 2013). Comparisons with an effect size (Cohen’s D) <0.8 were visualized separately to highlight near significant differences (Lakens, 2013) in order to screen for potential trends and identify regions with suggestive group differences that may warrant further consideration.

### 2.7 Constructing the functional connectome and network analysis

The c-Fos density for each counted nucleus was calculated by summing the cell counts from both hemispheres in bilateral regions, along with their respective area measurements, then dividing the cell number by the area (Stark et al., 2006; Stefaniuk et al., 2023). For the nuclei spanning multiple slides, the mean density was used.

**Figure 1:**
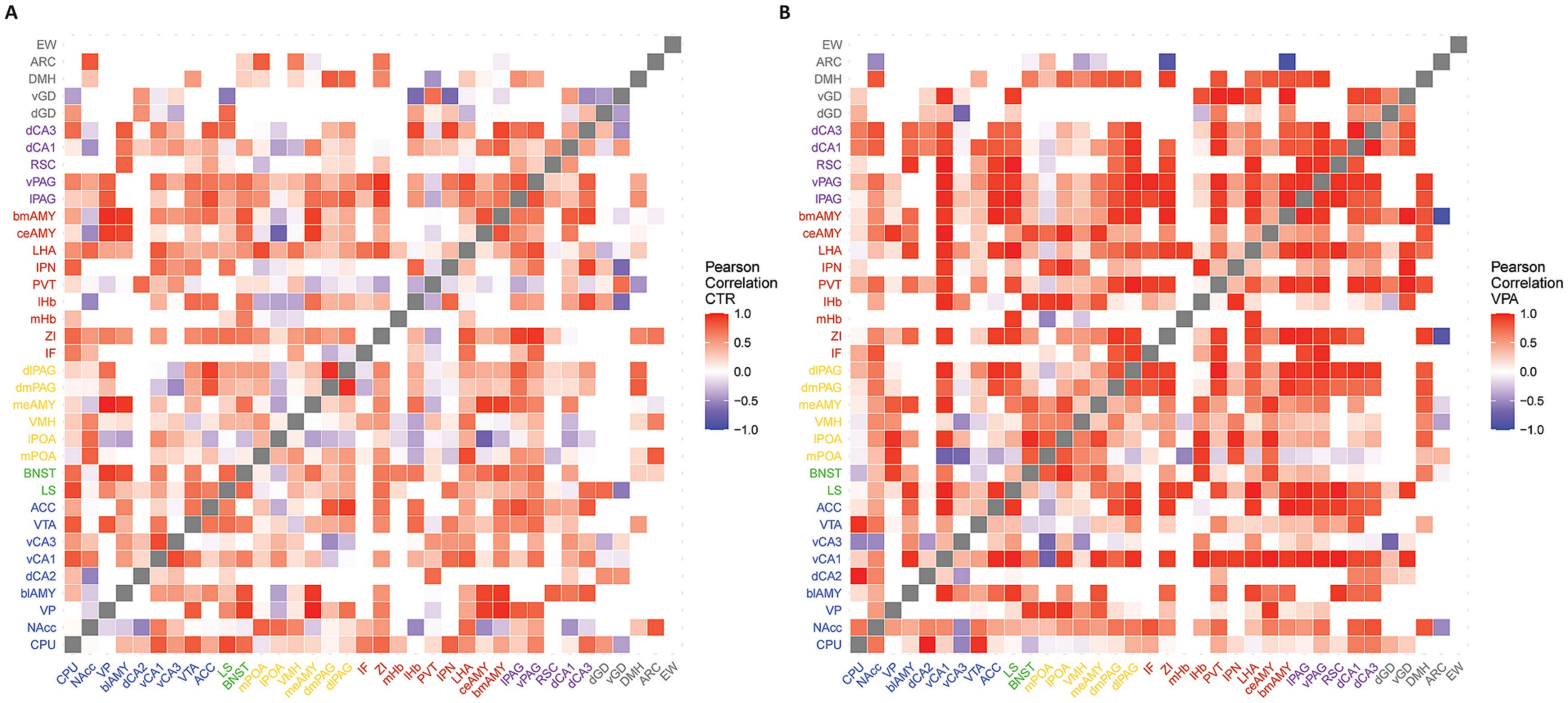
Heatmaps representing the functional connectivity between brain regions. The heatmaps depict the functional connectivity between brain regions, based on the correlation coefficients (R^2^, Pearson correlation) of their activation patterns. Red represents positive correlations, while blue indicates negative correlations. White squares represent no correlation or regions not connected physiologically according to the connectome used. **(A)** Heatmap representing the network of socially separated CTR animals. **(B)** Heatmap representing the network of socially separated VPA-treated mice.

**Figure 2:**
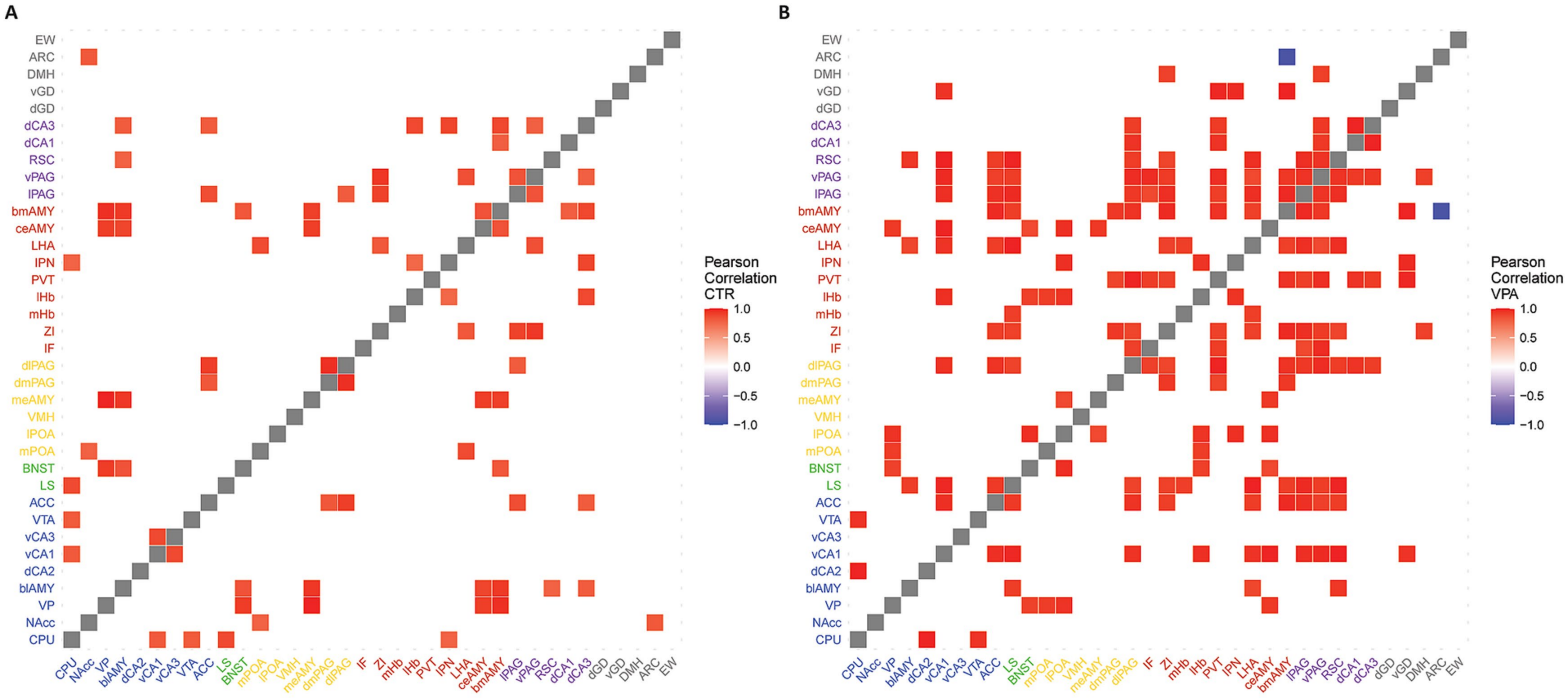
Heatmaps of functionally strongly connected areas. The heatmaps illustrate the functionally strongly connected brain regions. Functional connections were calculated based on the correlational strength of the activation in each nucleus. In the first step, the correlation matrices were thresholded using connectome data to reflect only physiologically connected areas. In the second step, matrices were further thresholded by correlational strength to display strong and significant connections (|R^2^| > 0.7, *p* < 0.05). Red areas indicate strong positive correlations, blue areas represent strong negative correlations, and white areas denote connections excluded either by the connectome thresholding or due to weak correlational strength. Panel **(A)** represents the heatmap of the CTR animals, while panel **(B)** shows the heatmap of the VPA-treated animals.

Within each of the two experimental groups (VPA, CTRL), all possible pairwise correlations between the Fos signal in the 36 brain regions were determined by computing Pearson’s correlation coefficients. The resulting *p*-values and R^2^ values were compiled into matrices. The R^2^ matrix was visualized as heatmaps (Figures 1, 2) using the “heatmaply” R-library (Galili et al., 2018).

**Figure 3:**
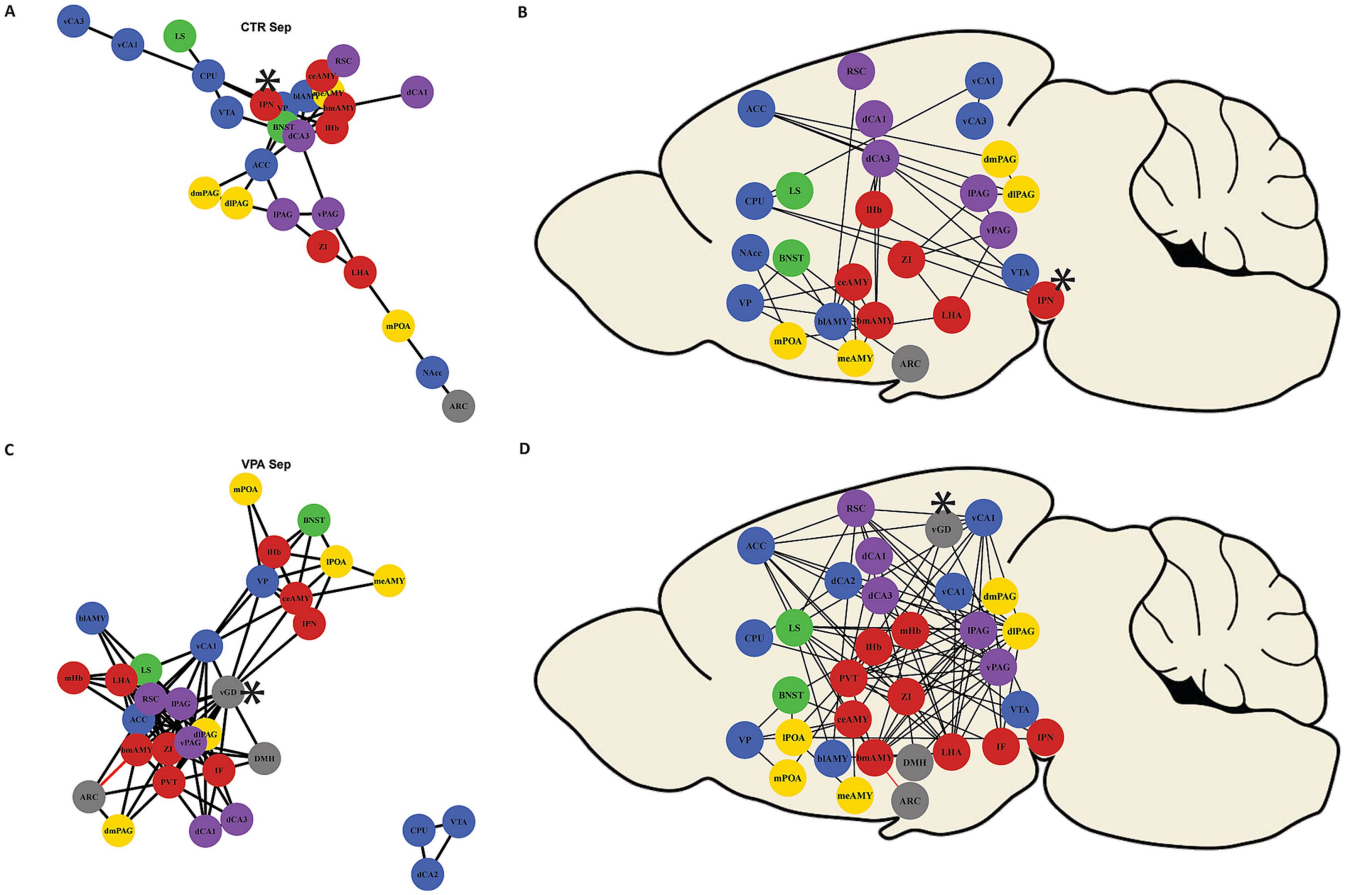
Network constructed by strong functional connections (|R^2^| > 0.7, *p* < 0.05). The network illustrates strong functional connections between brain regions. Black edges represent positive correlations, while red edges indicate negative correlations. Nodes represent the brain areas, and the colors of the circles correspond to the hypothesized subnetwork to which they belong (as shown in Figure 5). Panel **(A)** presents the functional network of control (CTR) mice. Panel **(B)** shows the connected nuclei in their approximate anatomical positions from a sagittal view. Panel **(C)** illustrates the more interconnected network of VPA-treated animals. Panel **(D)** represent the connected nuclei in their approximate anatomic position in the brain. Regions that had been identified as hubs (see text and Figures 6, 7) had been labeled by an asterisk.

For functional connectome network analysis, we used a methodology based on the work of Wheeler et al. (2013). To reconstruct the functional connectome, we included only R^2^ values that overlapped with connectivity data from the Allen Brain Atlas, ensuring that the heatmaps reflected physiologically plausible connections (Oh et al., 2014). This filtering step was crucial to eliminate indirect correlations, where two nuclei might exhibit similar activity not because of a direct anatomical connection, but due to a common upstream hub that synchronizes their activity. Our approach specifically aimed to identify these hub regions, as they play a key role in mediating large-scale network coordination. These values were thresholded on the basis of two criteria: an upper limit of *p* < 0.05 and a lower limit of R^2^ > 0.7. These correlations were considered as functional connections between brain regions and were visualized as edges in subsequent network visualizations using the igraph R-package (Csárdi et al., 2025). Black edges indicated positive correlations, while red edges indicated negative correlations (Figure 3).

For functional network analysis, degree and betweenness centrality values were calculated using the igraph package. Degree centrality quantifies how many direct connections (edges) a node has. A region with a high degree centrality is strongly connected to many other regions but does not necessarily act as a key relay in communication. Betweenness centrality, on the other hand, measures how often a node lies on the shortest path between other nodes. Betweenness centrality was calculated as B(*v*) = ∑[*σ_uw_*(*v*)/*σ_uw_*], where *σ_uw_* is the number of shortest paths between nodes *u* and *w*, and *σ_uw_*(*v*) is the number of shortest paths between *u* and *w* that pass through node *v*. The sum in the expression ranges over all pairs of distinct nodes *u* and *w* (Nguyen, 2014). A high betweenness score means that a node serves as a bridge, influencing how efficiently information flows through the network (Zhang and Luo, 2017; Kotlarz et al., 2024). For centrality analysis, a limit was defined at the 80% quantile of the sum of both centrality values. This criterion ensures that a region is not only highly connected (high degree) but also strategically positioned within the network, allowing it to act as a relay for indirect interactions. Nuclei ranking above the 80% quantile in both betweenness and degree centrality were classified as hubs.

## 3 Results

### 3.1 Three-chamber test

**Figure 4:**
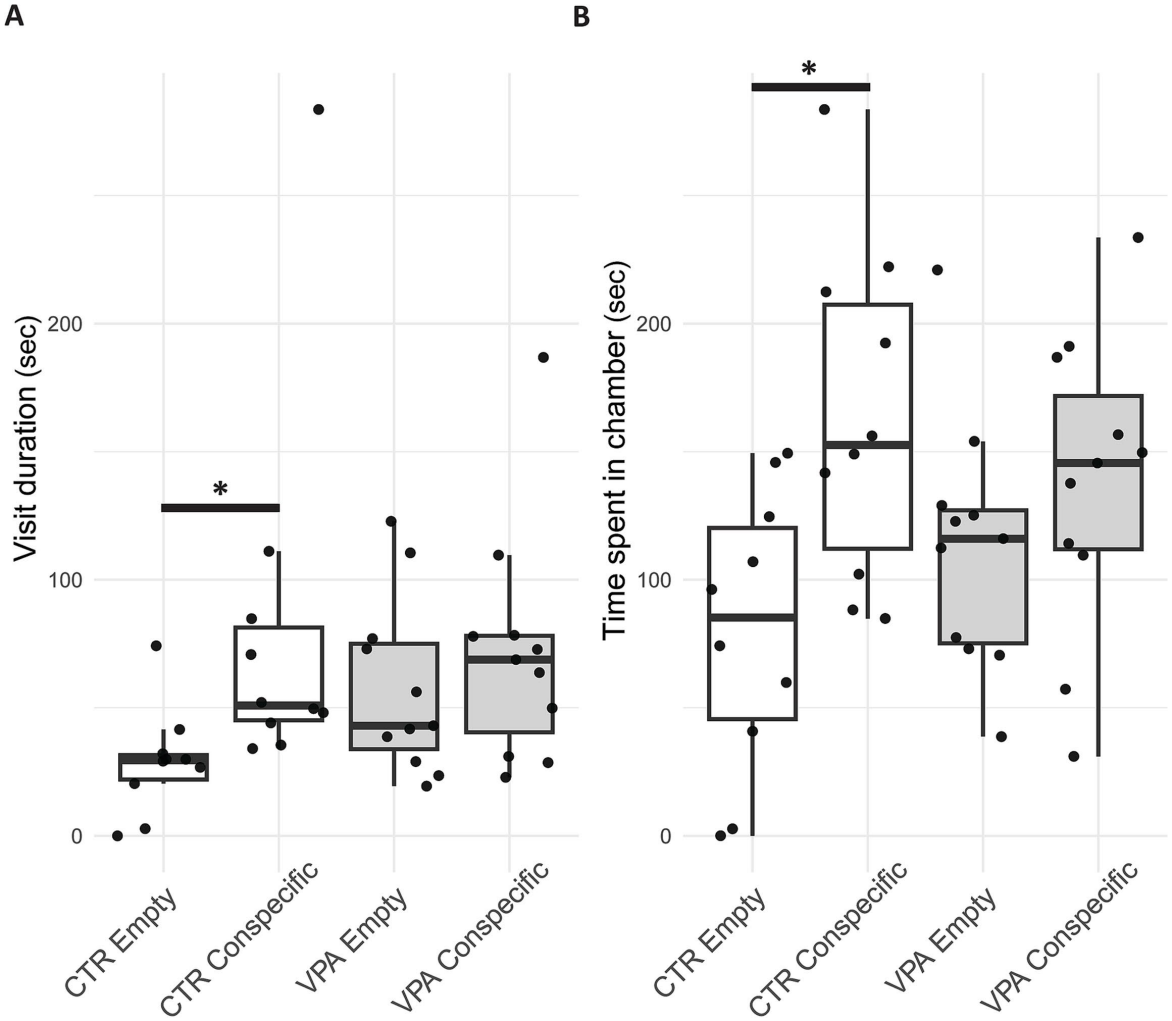
Boxplot of the three-chamber sociability test panel **(A)** shows the average time per visit (duration between the enter and exit into a chamber) spent by control (CTR) and valproate (VPA) treated mice in the chamber containing the conspecific (in a small cage) versus the chamber containing the empty cage. Panel **(B)** represents the total time spent by CTR and VPA-treated animals in the chamber with a conspecific compared to the empty chamber. Boxplot elements represent the median (horizontal line), interquartile range (box), min-max (whiskers) and outliers (dots outside whisker range).

In the control (CTR) group, animals spent significantly more time in the chamber containing an unfamiliar conspecific compared to the empty chamber [Z_(10)_ = −1.988, *p* = 0.047]. In contrast, there was no significant difference in chamber preference (Figure 4A) for the VPA-treated group [Z_(11)_ = −0.978, *p* = 0.328]. Regarding the average duration of visits (durations between the entry and exit into a chamber) in each chamber, CTR animals showed a more pronounced difference. The visits (Figure 4B) to the conspecific lasted significantly longer than those to the empty chamber [Z_(10)_ = −2.666, *p* = 0.008]. However, no such difference was observed in VPA-treated group [Z_(11)_ = −0.889, *p* = 0.374].

### 3.2 c-Fos activation patterns

**Figure 5:**
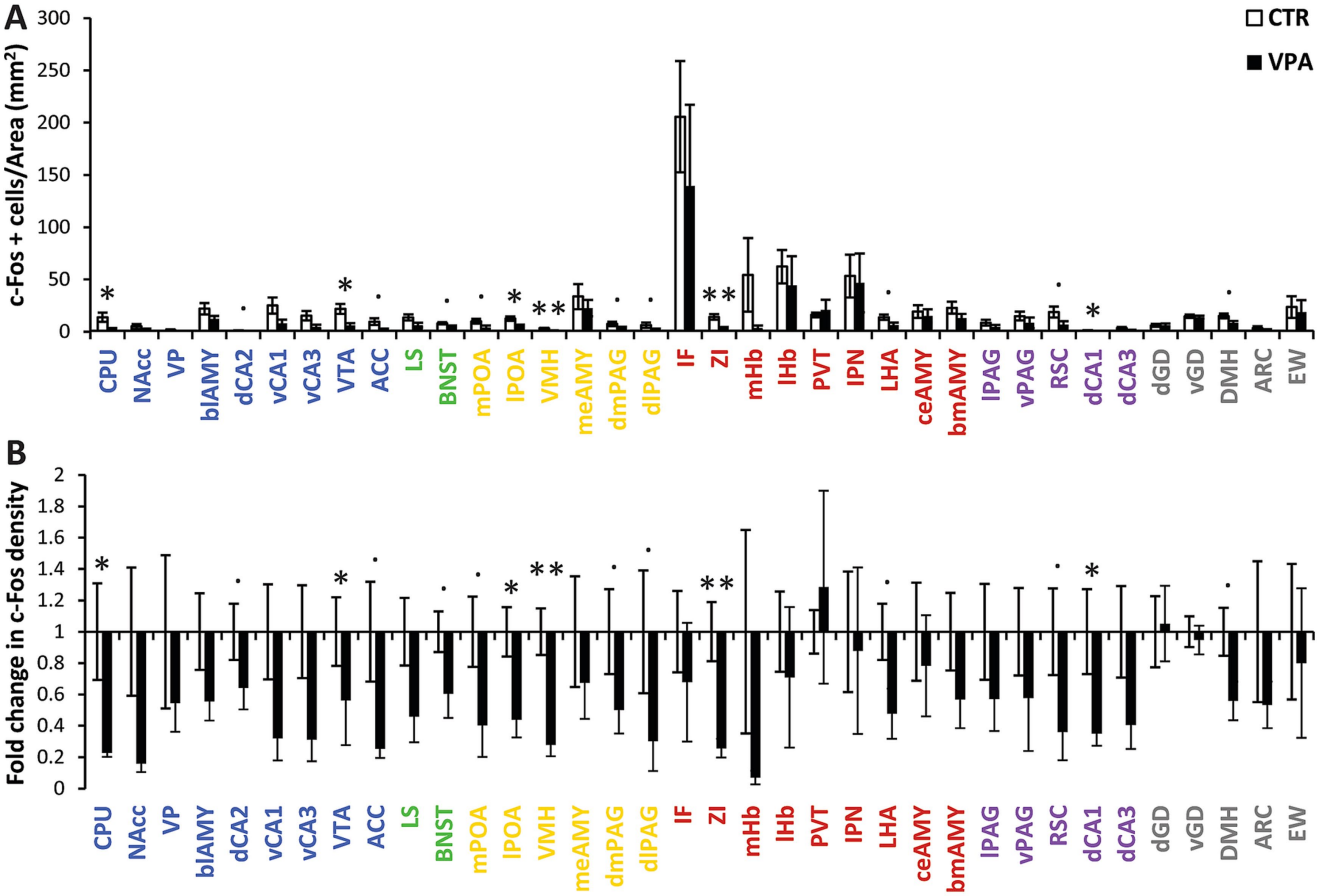
Activation patterns in regions of interest (ROIs) **(A)** the mean (± SEM) c-Fos density (number of c-Fos-positive cells per mm^2^) across different brain regions. The colors indicate the subnetworks to which the nuclei belong: blue represents the mesolimbic reward system (MRS), yellow represents the social behavior network (SBN), green represents overlapping nuclei that belong to both the MRS and SBN, red denotes nuclei involved in regulating stress (both social and non-social), purple represents regions not directly involved in social behavior but associated with autism-related activation or morphological differences, and gray represents control regions with vegetative functions or no described relation to social behavior or autism. **(B)** The same data are represented as fold change in c-Fos-positive cell density between control (CTR) and VPA-treated animals. The baseline value (1) represents the average activation density in the CTR group. Differences are indicated at three levels: a dot (.) represents marginal differences in activation density (0.05 < *p* < 0.1) with a high effect size (Cohen’s d > 0.8), a single asterisk (*) indicates significant differences (*p* < 0.05), and a double asterisk (**) indicates significant differences with adjusted *p* < 0.05 after FDR correction. Bars represent mean (± SEM).

Overall, the CTR group exhibited greater neural activity in response to social isolation compared to the VPA-treated group in the observed brain regions (Figure 5 and Supplementary Table S1). In all regions where group differences were detected, activity was consistently higher in the CTR group (Figure 5), and this trend was also apparent across regions qualitatively. Using a multivariate (multiple brain regions) mixed model on the separate networks c-Fos activity appeared significantly higher in the control animals in the MRS [*χ*^2^_(1, 279)_ = 4.463, *p* = 0.035]. A subsignificant trend was visible also in the SBN [*χ*^2^_(1, 191)_ = 3.719, *p* = 0.054]. A similar trend cannot be excluded even in the chosen control regions [*χ*^2^_(1, 90)_ = 2.83, *p* = 0.09], but there was no significant difference in the stress-regulating network regions [*χ*^2^_(1, 171)_ = 0.837, *p* = 0.360] (Table 1).

**Table 1:** Acronyms, full names, and network affiliations of brain nuclei analyzed in the experiment.

Acronym	Name	Network
CPU	Caudoputamen	Mesolimbic reward system (MRS)
NAcc	Nucleus accumbens	
VP	Ventral pallidum	
blAMY	Basolateral amygdala	
dCA2	Dorsal cornu ammonis 2	
vCA1	Ventral cornu ammonis 1	
vCA3	Ventral cornu ammonis 3	
VTA	Ventral tegmental area	
ACC	Anterior cingulate cortex	Extended MRS
LS	Lateral septum	Overlap between mesolimbic reward system and social brain network
BNST	Bed nucleus of stria terminalis	
mPOA	Medial preoptic area	Social brain network (SBN)
lPOA	Lateral preoptic area	
VMH	Ventromedial hypothalamus	
meAMY	Medial amygdala	
dmPAG	Dorsomedial periaqueductal gray	
dlPAG	Dorsolateral periaqueductal gray	
IF	Interfascicular nucleus	Stress (both social and non-social) regulating nuclei
ZI	Zona incerta	
mHb	Medial habenula	
lHb	Lateral habenula	
PVT	Paraventricular nucleus of the thalamus	
IPN	Interpeduncular nucleus	
LHA	Lateral hypothalamic area	
caAMY	Central amygdala	
bmAMY	Basomedial amygdala	
lPAG	Lateral periaqueductal gray	Regions not directly involved in social behavior but associated with autism-related activation
vPAG	Ventral peiaqueductal gray	
RSC	Retrosplenial cortex	
dCA1	Dorsal cornu ammonis 1	
dCA3	Dorsal cornu ammonis 3	
dGD	Dorsal dentate gyrus	Control regions
vGD	Ventral dentate gyrus	
DMH	Dorsomedial hypothalamus	
ARC	Arcuate nucleus	
EW	Edinger-Westphal nucleus	

Nuclei were categorized based on established functional network associations: mesolimbic reward system (MRS), social brain network (SBN), nuclei overlapping both MRS and SBN, regions involved in stress regulation (both social and non-social), regions not directly involved in social behavior but associated with autism-related activation, and control regions with vegetative functions or no documented relevance to social behavior or autism.

#### 3.2.1 Mesolimbic reward system

Activation patterns in the mesolimbic reward system are shown in blue in Figure 5. In CPU, VPA-treated animals exhibited significantly lower c-Fos activity compared to CTR animals [*χ*^2^_(1, 19)_ = 7.420, *p* = 0.006]. In the dorsal cornu ammonis 2 (dCA2), marginally higher activation of c-Fos was observed in the CTR group, with a large effect size [*χ*^2^_(1, 19)_ = 3.129, *p* = 0.077, Cohen’s d = 0.858]. A significant difference in activity was observed in the VTA, where the CTR group displayed greater activity than the VPA-treated group [*χ*^2^_(1, 19)_ = 7.027, *p* = 0.008]. In the ACC, marginally reduced activity was found in the VPA-treated group compared to the CTR group [*χ*^2^_(1, 42)_ = 3.127, *p* = 0.077, Cohen’s d = 1.137].

#### 3.2.2 Overlapping regions of the mesolimbic reward system (MRS) and social brain network (SBN)

Overlapping regions of the MRS and the SBN are indicated in green in Figure 5. In the BNST, there was a marginal difference between the activities in CTR and VPA-treated animals [*χ*^2^_(1, 39)_ = 3.286, *p* = 0.070], with a large effect size (Cohen’s d = 1.156). However, no significant difference was found between the two groups in the LS [*χ*^2^_(1, 19)_ = 1.712, *p* = 0.191].

#### 3.2.3 Social brain network

Activation patterns within the SBN are shown in yellow in Figure 5. Within the SBN, the mPOA showed marginally higher activity in CTR animals [*χ*^2^_(1, 18)_ = 3.176, *p* = 0.075, Cohen’s d = 1.111]. A similar but statistically significant difference was observed in the lPOA [*χ*^2^_(1, 19)_ = 7.089, *p* = 0.008]. VMH exhibited significantly higher activity in the CTR group, a result that remained significant after FDR correction [*χ*^2^_(1, 18)_ = 13.085, *p* < 0.001, FDR = 0.005]. Additionally, the dorsomedial (dmPAG) and dorsolateral periaqueductal gray (dlPAG), both being parts of the SBN, showed marginal differences in activity, with high effect sizes [dmPAG: *χ*^2^_(1, 8)_ = 3.728, *p* = 0.054, Cohen’s d = 0.840; dlPAG: *χ*^2^_(1, 19)_ = 3.350, *p* = 0.067, Cohen’s d = 0.827].

#### 3.2.4 Stress-regulating network

Activation patterns within the Stress-Regulating Network are shown in red in Figure 5. The ZI exhibited a significant difference between the treatment groups, remaining significant after FDR correction [*χ*^2^_(1, 17)_ = 14.922, *p* < 0.001, FDR = 0.004]. In the LHA, a marginal difference was found, with a large effect size [*χ*^2^_(1, 17)_ = 3.280, *p* = 0.070, Cohen’s d = 1.289].

#### 3.2.5 Other regions

Other analyzed brain regions not belonging to the predefined functional networks are shown in purple and grey in Figure 5. The retrosplenial cortex (RSC), a region associated with autism but not tightly linked to social behavior more like in salience processing of sensory inputs (Hogeveen et al., 2018; Shang et al., 2021; Shi et al., 2024), exhibited marginally higher activity in the CTR group [*χ*^2^_(1, 19)_ = 3.651, *p* = 0.056, Cohen’s d = 1.031]. Additionally, a significant difference was observed in the dorsal CA1 (dCA1) between CTR and VPA-treated groups [*χ*^2^_(1, 19)_ = 6.647, *p* = 0.001].

No significant differences were found in any other control regions, not implicated in social processing either. DMH showed a marginal difference, with lower activity in the VPA-treated group compared to the CTR group [*χ*^2^_(1, 17)_ = 3.506, *p* = 0.061, Cohen’s d = 1.323].

### 3.3 Functional connectome network analysis

To ensure that the correlative activation between brain regions (Figure 1), truly represents meaningful connections, we tested whether the region pairs with confirmed direct anatomical connections (based on the Allen Brain Atlas connectome [Oh et al., 2014)] exhibited higher correlations than those pairs without proven axonal connections (t = 2.601, df = 1952.1, *p* = 0.009) in the control mice. The correlations were higher between connected pairs than between unconnected pairs in the VPA animals, too (t = 19.157, df = 41.881, *p* < 0.001). The correlation matrices, based on known anatomical connections, and high (R^2^ > 0.7) and significant (*p* < 0.05) correlations (Figure 2), revealed a generally stronger co-activation of brain areas in VPA-treated animals as compared with CTR animals. When the network was reconstructed based on the matrices shown in Figure 2, distinct organizational differences were observed between VPA-treated and CTR groups when the correlation matrices were visualized as graphs (Figure 3). The VPA network exhibited a greater number of strong correlations, indicating broader functional connectivity across multiple brain regions. This suggests that VPA treatment alters network organization, leading to increased co-activation among several regions.

In CTR animals, the network was characterized by a structured and modular organization, with distinct clusters of nuclei and fewer interconnections (Figure 3A). A central group of stress-related regions was evident, while peripheral nodes maintained more selective connections. In contrast, the network in VPA-treated animals displayed a denser and highly interconnected structure, with more widespread links between regions (Figure 3B). This shift suggests a reorganization of functional connectivity, potentially reflecting altered information processing and integration across brain areas following VPA exposure.

**Figure 6:**
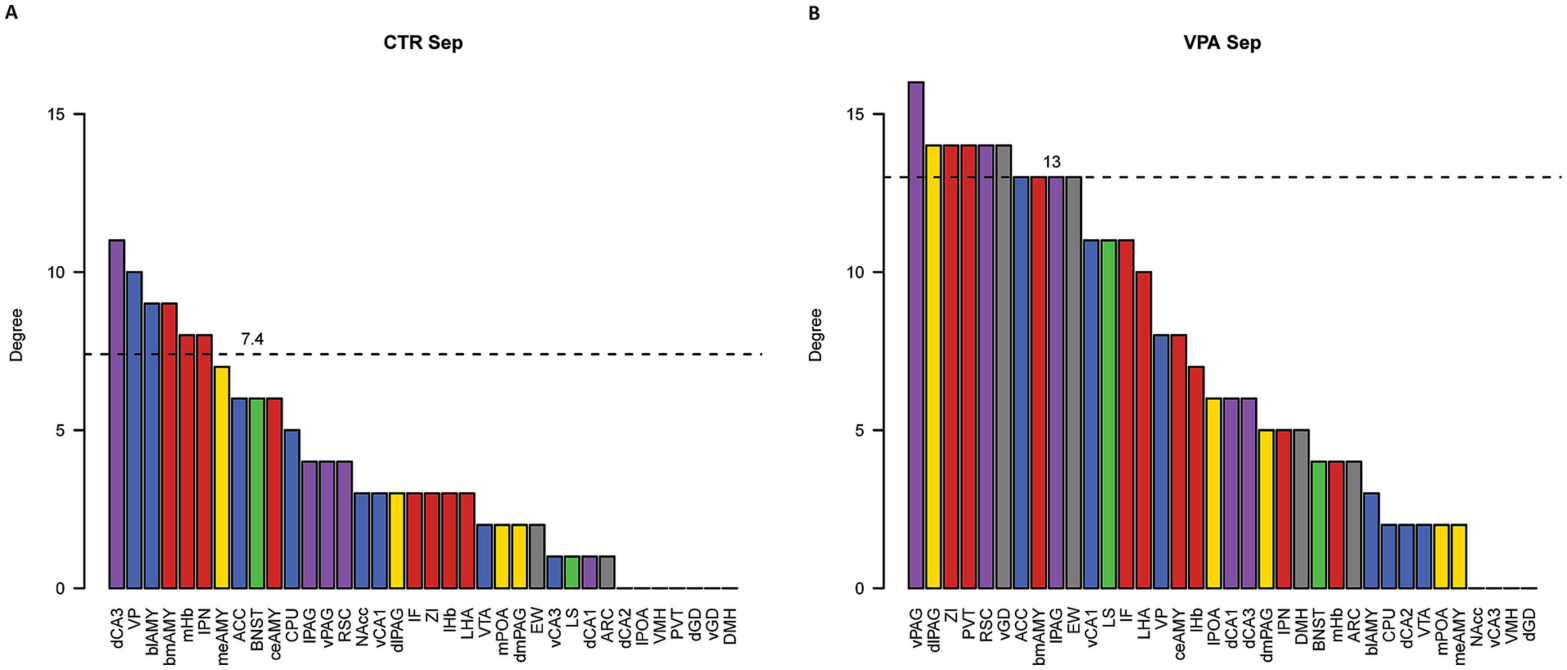
Degree centrality analysis of functional networks. Panel **(A)** shows the degree centrality values for control (CTR) mice, while panel **(B)** displays the values for VPA-treated mice. Degree centrality values represent the total number of edges connected to each nucleus. The dashed lines indicate the 80th percentile cutoff for centrality values, used to identify local hub nuclei with degree centrality values above this threshold. Bar colors indicate the subnetwork affiliations of the nuclei: blue represents the mesolimbic reward system (MRS), yellow represents the social behavior network (SBN), green represents overlapping nuclei belonging to both the MRS and SBN, red denotes nuclei involved in regulating stress (both social and non-social), purple represents regions not directly involved in social behavior but associated with autism-related activation or morphological differences, and gray represents control regions with vegetative functions or no observed changes related to social behavior or autism.

**Figure 7:**
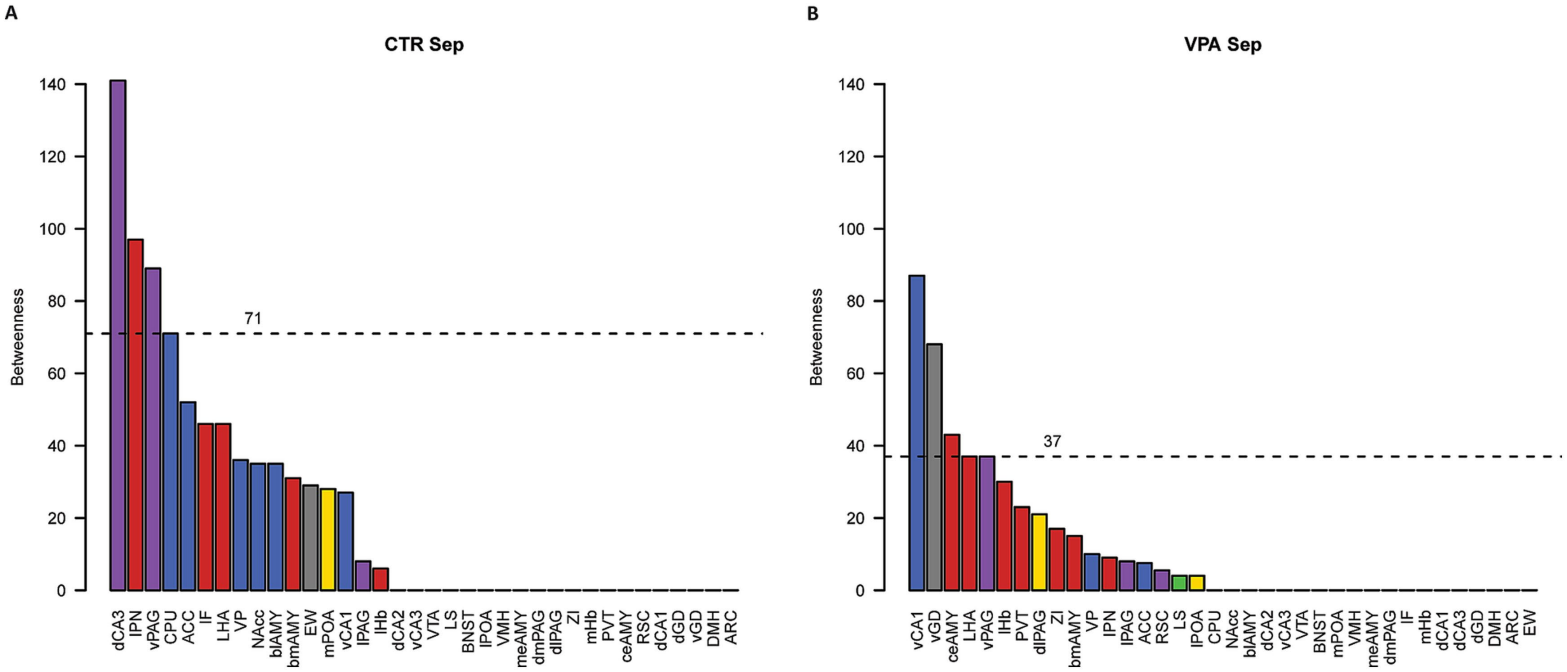
Betweenness centrality analysis of functional networks. Panel **(A)** shows the betweenness centrality values for control (CTR) mice, while panel **(B)** displays the values for VPA-treated mice. Betweenness centrality values indicate the central position of each nucleus within the network. The dashed lines represent the 80th percentile cutoff for centrality values, used to identify hub nuclei with high betweenness centrality values above this threshold. Bar colors indicate the subnetwork affiliations of the nuclei: blue represents the mesolimbic reward system (MRS), yellow represents the social behavior network (SBN), green represents overlapping nuclei belonging to both the MRS and SBN, red denotes nuclei involved in regulating stress (both social and non-social), purple represents regions not directly involved in social behavior but associated with autism-related activation or morphological differences, and gray represents control regions with vegetative functions or no observed changes related to social behavior or autism.

Quantitative network analysis revealed that, during social isolation, VPA-treated animals exhibited greater regional connectivity (degree centrality) compared to CTR animals (W = 376.5, *p* = 0.002) (Figure 6). Despite this increase in connectivity, the betweenness centrality, a measure of how crucial a node is in facilitating communication between different parts of the network (Wheeler et al., 2013; Zhang and Luo, 2017), did not differ significantly between the groups (W = 694.5, *p* = 0.572) (Figure 7). This suggests that, while VPA-treated animals had more connections overall, these connections did not increase the prominence of specific nodes as central hubs in the network.

Regions that displayed high ranks (above 80% of all ROIs) in both degree (Figure 6) and betweenness (Figure 7) centrality were considered as hubs within the observed brain network, as these regions were both highly connected and played a key role in mediating network-wide communication. In the CTR animals only IPN matched these criteria, ranking above the 80% threshold in both degree (Figure 6A) and betweenness centrality (Figure 7A). In contrast in VPA-treated mice, only the vGD exceeded the 80% threshold for both degree (Figure 6B) and betweenness centrality (Figure 7B). Overall, regions within the functional brain network of VPA individuals exhibited higher degree centrality (Figure 6) but similar—or even lower, considering the highest quantiles—betweenness centrality (Figure 7) compared to CTR mice.

## 4 Discussion

The present study provides critical insights into the distinct neural activation patterns observed in adolescent (31-day-old) male VPA-treated animals, a widely recognized model for ASD, under conditions of social isolation. Using a network-based, activity-dependent approach, we identified significant alterations in both regional activation and functional connectivity compared to CTR animals, highlighting widespread disruptions in the neural mechanisms underlying the social deficits commonly associated with ASD.

Looking at the activation patterns, it was evident that social isolation had a stronger impact on CTR animals than on VPA-treated animals, as the latter exhibited lower activation in several of the observed nuclei and also in some of the functional networks. The regions with higher activation in CTR animals are not only involved in modulating social behaviors but also play crucial roles in decision making, often driven by social stimuli.

Examining c-Fos expression revealed region-specific differences in neural activation between VPA-treated and CTR animals following social isolation. The most striking changes were observed in two significant regions after FDR adjustment: the VMH and ZI. The VMH, a core component of the SDMN (O’Connell and Hofmann, 2011), plays a crucial role in defensive behaviors and social stress responses. Increased c-Fos expression in the VMH of CTR animals suggests that they perceived social isolation as a stressor, aligning with its role in regulating avoidance and defensive behaviors (Bandler and Keay, 1996; Shao et al., 2023). In contrast, the significantly lower VMH activation in VPA-treated animals implies a blunted stress response, supporting the hypothesis that these animals experience social separation differently. Another differentially activated region, the ZI, is a key stress regulatory nucleus that integrates sensory and limbic information to modulate defensive behaviors and emotional responses. Its heightened activation in CTR animals is consistent with its role in initiating stress-adaptive mechanisms, particularly in regulating arousal and coping strategies under socially challenging conditions (Ahmadlou et al., 2021). In contrast, the diminished ZI activity in VPA-treated animals suggests an impaired capacity to process stress, which may contribute to their altered behavioral responses. The ZI has been implicated in reinforcement learning and behavioral flexibility, particularly in modulating responses to novel stimuli (Monosov et al., 2022). Importantly, specific subpopulations of ZI neurons have been shown to encode and regulate different components of anxiety, further reinforcing their role in emotional regulation (Li et al., 2021). The reduced engagement of this region in VPA-treated animals may thus reflect a diminished ability to modulate anxiety-related responses, which could underlie the atypical stress processing observed in ASD models.

Beyond the FDR-corrected significant findings, several additional SDMN-associated regions displayed significantly higher activation in CTR animals, though they did not survive multiple comparison corrections but had considerable effect size. The VTA, a key dopaminergic center critical for motivation and reward processing, showed elevated activation in CTR animals, suggesting increased social drive, consistent with its role in regulating social approach behaviors (Schultz, 1998; Kinard et al., 2020). Stress-related social behaviors are also linked to specific neuronal ensembles within the VTA, emphasizing its dual role in stress and reward processing (Koutlas et al., 2022). Similarly, the CPU, which is involved in social stimulus processing and goal-directed actions, exhibited enhanced activation in CTR animals, suggesting a stronger motivation to restore social contact (Wickens et al., 2007; Contesse et al., 2021). Another key SDMN region, the lPOA, contributes to social behaviors by regulating reward-driven social motivation and approach behaviors. Its heightened activation in CTR animals further supports the idea of an enhanced social drive in response to isolation (O’Connell and Hofmann, 2011). Notably, the lPOA modulates dopaminergic signaling via VTA disinhibition, facilitating goal-directed behavior and adaptive responses following aversive experiences (Gordon-Fennell et al., 2020). Additionally, the dCA1 of the hippocampus showed significant activation in CTR animals. While dCA1 is not classically associated with social behavior, it is implicated in cognitive flexibility and contextual memory processing, functions that may contribute to stress adaptation. Aberrant dCA1-VTA hyperconnectivity has been implicated in psychiatric conditions such as schizophrenia and ASD, where hippocampal dysregulation alters dopamine-related behavioral responses (Zhang et al., 2022).

A set of additional brain regions exhibited activation trends, though they did not reach significance. Most of these regions are part of either the SDMN or the social stress network, and their altered activation patterns further support the notion that CTR animals engage a broader social and stress-related network compared to VPA-treated animals. Among these, the dCA2 plays a critical role in social memory encoding and recall, strongly influencing ventral CA1, which contains neurons implicated in social memory processing (Meira et al., 2018). The ACC, which plays a crucial role in social cognition and reward prediction, is particularly involved in decision-making processes that require physical and emotional effort but not in all forms of cost–benefit decision-making (Wang et al., 2017). The BNST, which is essential for social recognition and context assessment, integrates sensory and emotional cues about conspecifics and plays a pivotal role in processing social stress and modulating long-term social behaviors (Flanigan and Kash, 2022). The dmPAG and dlPAG also showed activation trends, with the dmPAG modulating aggressive behaviors, receiving inputs from the VMH, a well-known aggression center (Li et al., 2025), while the dlPAG is associated with fear conditioning and emotional memory processing, regulating adaptive responses to environmental challenges (Kincheski et al., 2012).

The LHA, known to play a role in social stress responses, has been linked to defensive behaviors through glutamatergic activity (Li et al., 2018). Though not part of the SDMN, the RSC is critical for spatially-guided social behavior, responding to social novelty and processing environmental-social interactions (Yamawaki et al., 2019; Shang et al., 2021). While traditionally viewed as a region unrelated to social behavior, the DMH also plays a role in emotional regulation and adaptive stress responses, with lower activation in VPA-treated animals suggesting an overall blunted stress response (DiMicco et al., 2002; Kataoka et al., 2013). The differential activation of the regions presumably unrelated to the SDMN suggested that the effect of VPA is most likely not limited to the social brain, and to identify the real functional changes, one should not pinpoint one or a few brain regions or even pathways, but to analyze the wide functional network.

Network reconstruction revealed distinct organizational patterns between CTR and VPA-treated animals, particularly in terms of functional connectivity hubs. In CTR animals, the IPN emerged as a central hub within the social stress regulatory network, forming a strong connection with the lHb (Klenowski et al., 2023). This IPN-centered network integrates stress responses with motivational and social processing circuits, emphasizing the IPN-lHb axis as a key regulatory pathway in response to social isolation (Sugama et al., 2002; Benekareddy et al., 2018; Ogawa and Parhar, 2022). The lHb, in particular, is known to process aversive social stimuli and regulate avoidance behavior (Hikosaka, 2010; Cobb-Lewis et al., 2024), while the mHb, though not as centrally connected as the lHb, is implicated in chronic stress adaptation and depressive-like states (Xu et al., 2018). The strong connectivity between the IPN and lHb observed in CTR animals aligns with previous findings on the IPN-lHb axis, which plays a crucial role in coordinating adaptive stress responses under socially challenging conditions.

In contrast, VPA-treated animals lacked a strong IPN-centered network, with vGD emerging as the only hub. However, the vGD (and every other regions) in the VPA-treated mice exhibited much lower betweenness centrality compared to IPN in controls, indicating that VPA-treated animals engage a less efficient, more fragmented functional network. These findings suggest fundamental alterations in how VPA-treated animals process social isolation, with diminished stress and reward circuit engagement, aligning with behavioral deficits observed in ASD models. Reduced activation of SDMN and stress-regulatory regions, including the VMH, ZI, and IPN, implies diminished motivation to reinstate social contact. Moreover, the loss of IPN as a central hub suggests that social isolation may not be perceived as equally stressful by VPA-treated animals, further contributing to weaker motivation to avoid separation.

The present study is the first to systematically map the neural activation, measured by c-Fos expression, of animals with autistic phenotypes during social isolation. It is clear from the differential activation that the abnormal brain development caused by the embryonic VPA treatment is not restricted to a single or a few brain regions. Such network-wide effects were also supported by our qualitative and quantitative network analysis: The VPA mice exhibit a more interconnected functional connectome than controls, albeit with a lower betweenness centrality, suggesting that many of the key regions lose their hub-like function in the processing of social separation.

These results align with behavioral evidence demonstrating reduced sociability, lower vocalization, and diminished social play in VPA models (Servadio et al., 2018; Tartaglione et al., 2019; Chaliha et al., 2020). Impaired reward and stress circuit engagement in VPA animals likely underlies these social deficits, mirroring clinical observations in ASD (Grace et al., 2022). Crucially, these findings suggest that it is not only the response to social stimuli that is altered in ASD but also the fundamental drive to avoid social isolation. Understanding these mechanisms is essential for developing interventions that restore social motivation in autism, shifting the focus not only to altered responses to social stimuli but also to the underlying drive for social contact.

Our findings suggest that VPA-treated animals may perceive social isolation as less stressful due to disruptions in the neural circuits responsible for processing stress, reward, and social stimuli. Future studies should focus on further investigation into wider network-level differences to develop targeted interventions aimed at improving social behavior in individuals with ASD.

### 4.1 Limitations of the study

While the method of reconstructing functional networks based on cFos activation does not replace direct circuit-tracing approaches, such as fMRI, optogenetics or electrophysiological recordings, recent research supports the use of c-Fos expression patterns to infer large-scale functional networks. Stefaniuk et al. (2023) demonstrated that c-Fos-based network analyses can accurately capture behaviorally relevant functional connectivity, while Kotlarz et al. (2024) further confirmed its usefulness in identifying experience-dependent neural network changes. Additionally, Stark et al. (2006) compared c-Fos activity patterns with fMRI-based functional connectivity, concluding that c-Fos mapping provides highly reliable spatial resolution of functionally active circuits. The possibility cannot be excluded that brain regions without direct anatomical connections could exhibit similar Fos activation patterns due to shared functional roles or parallel processing of social stress responses. By including only neuroanatomically verified connections, we aimed to reduce spurious correlations and highlight the differences in the network structure. This approach helps mitigate the risk of falsely interpreting activity synchronization between two nuclei as a direct functional link when, in reality, their activity is driven by a third common hub. Furthermore, our methodology was specifically intended to identify these hub regions, as they are the critical mediators of network-wide coordination.

Research suggests that females may be more resilient to prenatal VPA exposure due to compensatory mechanisms, including hormonal modulation and alternative social coping strategies, which make ASD-like traits less evident in early developmental stages (Lai et al., 2013; Hull et al., 2020). In juvenile rodents, females often display more adaptive social behaviors, potentially masking the full impact of prenatal VPA exposure (Hiller et al., 2014; Ratto et al., 2018). This aligns with human studies showing that autistic females are often diagnosed later than males, as their behavioral phenotypes diverge from the classical, male-based diagnostic framework (Lai et al., 2011; Belcher et al., 2023). Given these sex differences, our study specifically aimed to investigate the classical ASD-like phenotype, which is more reliably expressed in male subjects during juvenile stages. While the question of sex differences in ASD is a worthy topic of research, it was beyond the scope of the present study. Future work incorporating both sexes, particularly at later developmental stages, may provide further insights into sex-specific ASD manifestations.

## Data Availability

The raw data supporting the conclusions of this article will be made available by the authors, without undue reservation.

## Supplementary material

Supplementary Figure 1 The planes from which the sections were collected. The original atlas pictures are from the Allen Brain Atlas (ISH Data: Atlas Thumbnails, 2024). The colors of the regions indicate the subnetwork affiliations of the nuclei: blue represents the mesolimbic reward system (MRS), yellow represents the social behavior network (SBN), green represents overlapping nuclei belonging to both the MRS and SBN, red denotes nuclei involved in regulating stress (both social and non-social), purple represents regions not directly involved in social behavior but associated with autism-related activation or morphological differences, and gray represents control regions with vegetative functions or no observed changes related to social behavior or autism.

Supplementary Figure 2 Representative photomicrographs of c-Fos labeled sections. Panel **(A)** shows a slice from a CTR animal. Panel **(B)** is a sample taken from VPA-treated animal. Dashed lines delineate some regions of interests. Arrowheads point to c-Fos labeled nuclei.
