# Supplementary figures and images for: Disrupted functional connectome in a rodent model of autism during social isolation
Source: Front Neural Circuits. 2025 May 14;19:1525130. doi: 10.3389/fncir.2025.1525130 (PMC12116437; doi:10.3389/fncir.2025.1525130)

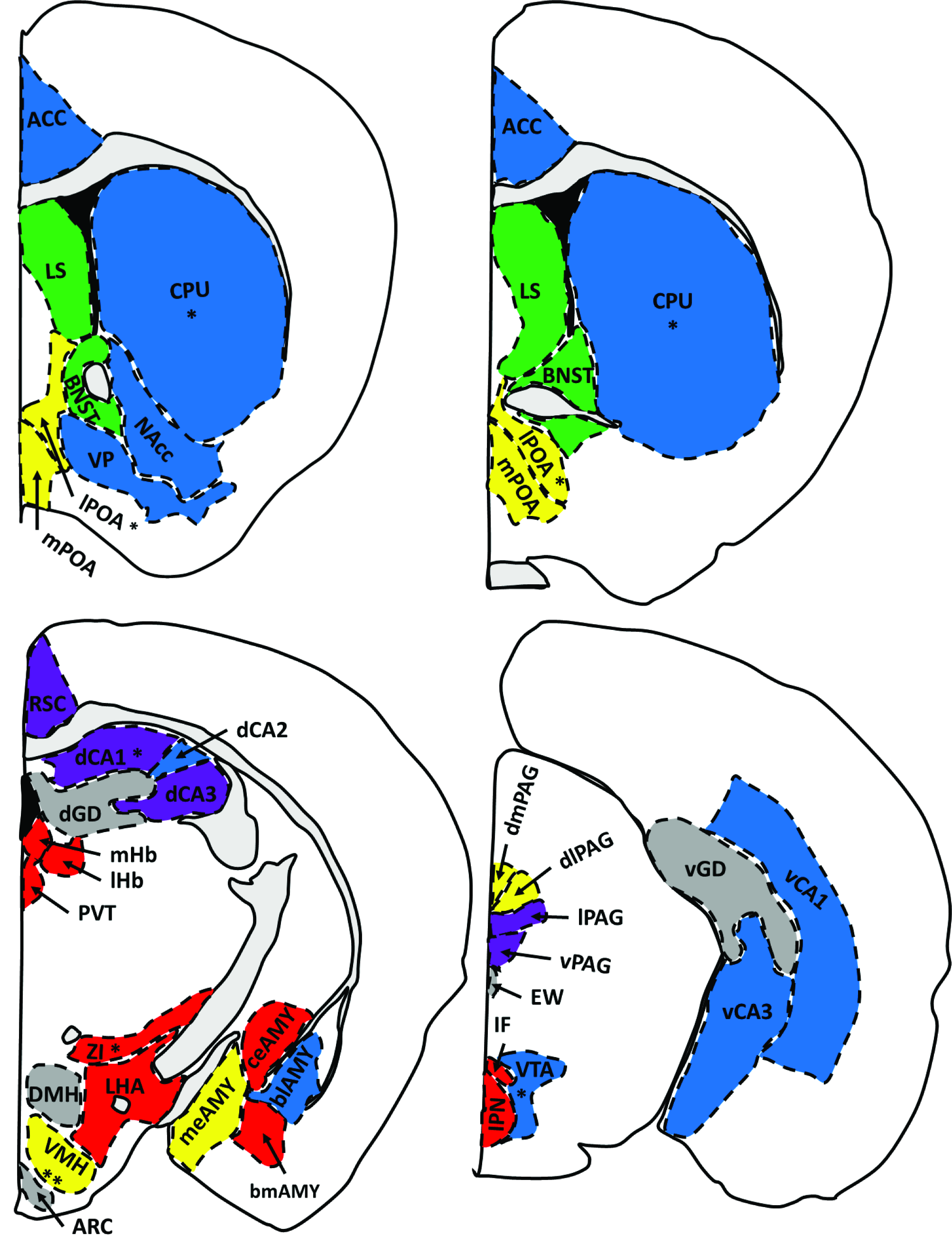

Supplement: Supplementary Figure 1 — The planes from which the sections were collected. The original atlas pictures are from the Allen Brain Atlas (ISH Data: Atlas Thumbnails, 2024). The colors of the regions indicate the subnetwork affiliations of the nuclei: blue represents the mesolimbic reward system (MRS), yellow represents the social behavior network (SBN), green represents overlapping nuclei belonging to both the MRS and SBN, red denotes nuclei involved in regulating stress (both social and non-social), purple represents regions not directly involved in social behavior but associated with autism-related activation or morphological differences, and gray represents control regions with vegetative functions or no observed changes related to social behavior or autism. [file Image_1.tif]

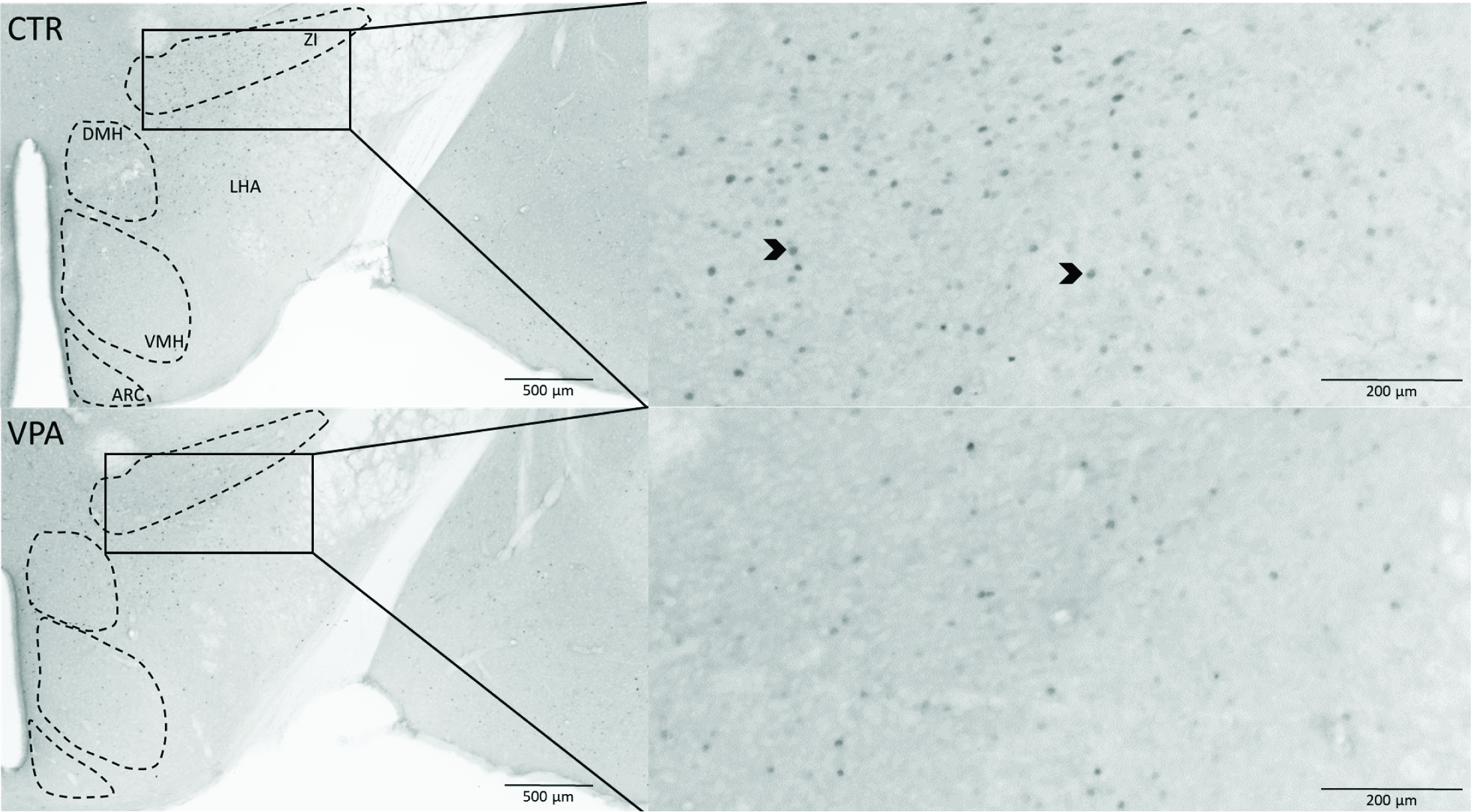

Supplement: Supplementary Figure 2 — Representative photomicrographs of c-Fos labeled sections. Panel (A) shows a slice from a CTR animal. Panel (B) is a sample taken from VPA-treated animal. Dashed lines delineate some regions of interests. Arrowheads point to c-Fos labeled nuclei. [file Image_2.tif]
